# Clinical outcomes of tebentafusp in metastatic uveal melanoma: a systematic review and single-arm meta-analysis

**DOI:** 10.3389/fmed.2026.1826626

**Published:** 2026-06-17

**Authors:** Han Cai, Zhilong Huang, Bing-Long Wang, Ming Wei, Miao Tian, Hongmei Zheng

**Affiliations:** 1 Ophthalmology Center, Renmin Hospital of Wuhan University, Wuhan, Hubei, China; 2 Guangzhou Medical University, Guangzhou, China; 3 School of Health Policy and Management, Chinese Academy of Medical Sciences, Peking Union Medical College, Beijing, China

**Keywords:** meta-analysis, metastatic uveal melanoma, tebentafusp, trials, tumor - classifications & detections

## Abstract

**Objective:**

To evaluate efficacy and safety of tebentafusp in patients with metastatic uveal melanoma.

**Method:**

A systematic search was conducted in five databases (Pubmed, Embase, Web of Science, Scopus, and the Cochrane Library) to find articles that evaluate the effectiveness of tebentafusp in patients with metastatic uveal melanoma, from the establishment of the databases to October 13, 2025. Additionally, clinical trials were searched on clinicaltrials.gov. Meta-analyses were performed to evaluate the frequencies of progression-free survival (PFS), overall survival (OS), partial response (PR), stable disease (SD), progressive disease (PD), objective response rate (ORR), disease control rate (DCR), Cytokine release syndrome (CRS) and treatment-related adverse events (trAEs).

**Results:**

A total of six studies were initially enrolled, involving 850 participants. Among them, 631 patients with evaluable uveal melanoma who received tebentafusp treatment were ultimately included in this meta-analysis (mean age, 60.56 years; 425 [50%] men). The median OS and PFS were 21.3 months and 3.6 months, respectively. The PR, SD, PD,ORR and DCR were 0.09 [95% confidence interval (CI), 0.05 to 0.14], 0.39 (95% CI, 0.35 to 0.43), 0.79 (95% CI, 0.32 to 0.97), 0.09 (95% CI, 0.06 to 0.13) and 0.27 (95% CI, 0.20 to 0.36), respectively. The any grade cytokine release syndrome rate, grade ≥ 3 cytokine release syndrome rate, any grade trAEs rate and grade ≥ 3 trAEs rate were 0.88 (95% CI, 0.84 to 0.91), 0.02 (95% CI, 0.01 to 0.06), 0.98 (95% CI, 0.75 to 1.00) and 0.41 (95% CI, 0.17 to 0.71), respectively. The most common adverse reactions include pyrexia 0.83 (95% CI, 0.79 to 0.86), nausea 0.58 (95% CI, 0.42 to 0.73), fatigue 0.54 (95% CI, 0.38 to 0.70), pruritus 0.72 (95% CI, 0.65 to 0.78), chills 0.60 (95% CI, 0.47 to 0.71), dry skin 0.43 (95% CI, 0.25 to 0.63), hypotension 0.41 (95% CI, 0.36 to 0.45).

**Conclusion:**

The research results show that tebentafusp exhibits modest but potentially meaningful survival benefit despite low response rates for metastatic uveal melanoma patients. Although adverse events often occur, such as cytokine release syndrome of grade three or above, they are usually manageable and controllable. Given the limitations of this study, it is crucial to conduct further multi-center randomized controlled trials and increase the sample size to further verify our results.

**Systematic review registration:**

PROSPERO: CRD420251234508. Available from https://www.crd.york.ac.uk/PROSPERO/view/CRD420251234508.

## Introduction

Uveal melanoma (UM) is the most common primary intraocular malignancy in adults (1). Its pathophysiological profile differs substantially from that of cutaneous melanoma (2, 3), notably lacking common driver mutations such as BRAF and NRAS (4–6). Instead, UM is molecularly characterized by Gαq/11 subunit-related gene mutations (present in >80% of cases) and inactivating BAP1 mutations (≈50%, associated with poor prognosis) (6, 7).

Globally, the incidence of UM ranges from 0.1 to 8.6 per million population, with a higher incidence at northern vs. southern latitudes. Approximately 98% of affected individuals are of Caucasian ethnicity, and the long-term incidence trend remains stable (8–13). In 2023, 3,490 new UM cases were reported in the United States (14–16). Although only 4% of patients have metastases at initial diagnosis, up to 50% eventually develop distant metastases-−90% involving the liver—and 60% of recurrences occur within five years (14, 17–20). Metastatic UM (mUM) carries an extremely poor prognosis: historical data indicate a median overall survival (mOS) of 10–13 months and a 5-year survival rate of only 16% (21–23). Among untreated primary UM patients, disease-specific survival declines sharply from 71% at 5 years to 29% at 10 years, a stark contrast to outcomes in treated cohorts (24). Molecular classification based on gene expression profiles (GEP) and chromosomal copy-number alterations identifies high-risk UM as the Class 2 subtype, driven by BAP1 mutations and deletions of chromosomes 1p, 3, 6q, and 8p. This subtype is distinguished by an immunosuppressive tumor microenvironment and a high metastatic propensity, in contrast to the lower-risk Class 1 subtype defined by EIF1AX or SF3B1 mutations (7).

Local treatment of UM has gradually evolved from traditional enucleation to eye-sparing precision radiotherapy (17). Currently, iodine-125 brachytherapy and proton radiotherapy are the mainstream modalities for early localized UM, achieving local control rates of up to 95% (25, 26). For large or locally advanced tumors, enucleation remains indispensable (14, 27). While local therapy effectively controls the primary tumor, it does not reduce the risk of distant metastasis; approximately 50% of patients eventually progress to mUM (14, 18, 28, 29). Conventional chemotherapies—such as dacarbazine and fotemustine—have limited efficacy, with objective response rates (ORR) of 0–15% and an mOS of < 12 months, offering no meaningful survival benefit (1, 19, 30). Although combined anti-PD-1/CTLA-4 therapy yields a higher ORR (18%) in mUM than monotherapy (ORR 0–3.6%), its utility is constrained by a high incidence of grade 3–4 adverse events (up to 40.4%) and an efficacy still substantially inferior to that observed in cutaneous melanoma (31, 32). This limited response is attributed to the low tumor mutational burden (TMB) of mUM, low PD-L1 expression, and an immunosuppressive microenvironment characterized by CD8+ T-cell exhaustion, where these T cells predominantly express LAG3 rather than PD-1 or CTLA-4 (7, 33–38). Given the rarity of BRAF mutations in UM, traditional tyrosine kinase inhibitors (TKIs) show limited activity; for instance, selumetinib combined with chemotherapy failed to improve progression-free survival (PFS) (39–42).

Tebentafusp—a first-in-class HLA-A^*^02:01-restricted ImmTAC bispecific fusion protein—activates T cells by targeting the gp100 antigen via a high-affinity T-cell receptor (TCR) and engaging CD3. This mechanism led to FDA and EMA approval in 2022 for the treatment of mUM (14, 43–45). Phase III trials have demonstrated that tebentafusp significantly extends mOS to 21.7 months, compared with 16.0 months for conventional therapy, establishing it as the first systemic agent proven to improve survival in Mum (46). Previous meta-analyses (47, 48) included a limited number of studies and did not exclude retrospective data. Therefore, an updated meta-analysis incorporating the latest clinical trial results is warranted to provide high-level evidence for the rational sequencing and selection of tebentafusp in clinical practice.

## Material and methods

### Search strategy

This meta-analysis was conducted in accordance with the 2020 Preferred Reporting Items for Systematic Reviews and Meta-Analyses (PRISMA) guidelines (49). The meta-analysis has been registered with PROSPERO under registration number CRD420251234508. A comprehensive literature search was conducted across five electronic databases (PubMed, Embase, Web of Science, Scopus, and the Cochrane Library) for studies published up to October 13, 2025. Additionally, clinical trials were identified through searches on clinicaltrials.gov. The search strategy adhered to the PICOS (Population, Intervention, Comparison, Outcomes, Study design) framework and incorporated a combination of MeSH terms and free-text keywords.

The specific search strategy used was: “Tebentafusp”. The detailed search strategy for all databases is provided in Supplementary Table 1.

### Inclusion and exclusion criteria

Inclusion criteria were as follows: (1) Patients diagnosed with metastatic uveal melanoma; (2) Patients in the intervention group received tebentafusp; (3) Patients in the controlled group received active comparators or placebo; (4) Outcome focused on safety and efficacy data.; (5) Study types: Clinical trials.

The exclusion criteria are as follows: (1) Duplicate literature; (2) Other types of articles, such as case reports, publications, thesis, letters, comments, reviews, meta-analyses, editorials, protocols, retrospective study, etc; (3) Not relevant; (4) The safety and efficacy data were not reported.

### Selection of articles

The literature selection process was managed using EndNote (Version 20; Clarivate Analytics) to identify and remove duplicate records. Two independent reviewers then screened the titles and abstracts of the remaining records for relevance. For studies undergoing full-text review, if safety and efficacy data were not available in the main text, we examined Supplementary materials. We also queried the ClinicalTrials.gov database using the provided trial registration identifiers (e.g., NCT numbers) to locate any reported outcomes. Any disagreements between the reviewers were resolved by consensus, with a third author acting as arbiter when necessary.

### Data extraction

The data were extracted independently by two reviewers using standardized forms. The extracted information included: (1) Basic study characteristics, including NCT number, study design, and sample size; (2) Baseline demographics and clinical characteristics of participants, including patient numbers, population, age, treatment regimens, and follow-up duration; (3) Safety and efficacy outcomes. Any discrepancies were resolved through consultation with a third investigator.

### Quality assessment

The risk of bias in the included studies was assessed by two independent reviewers. Randomized controlled trials were evaluated using the modified Jadad scale, while single-arm trials were appraised with the Methodological Index for Non-Randomized Studies (MINORS) criteria (50). Any discrepancies in assessment were resolved through group consensus.

### Statistical analysis

All statistical analyses were performed using R software (version 4.5.2) within RStudio (2025.09), primarily leveraging the meta package. For time-to-event outcomes, individual patient data were reconstructed from Kaplan-Meier curves using the GetData Graph Digitizer software, followed by application of the IPDfromKM utility, which implements the method described by Guyot et al. (51).

Individual patient data were reconstructed from published Kaplan-Meier survival curves using the IPDfromKM method. First, coordinate points (time and survival probability) were digitally extracted from each curve. Second, data preprocessing was performed, including sorting by time, monotonicity adjustment to ensure non-increasing survival over time, and outlier removal using Tukey's fence. Third, the modified iterative Kaplan-Meier (modified-iKM) algorithm was applied to estimate the number of events, censored cases, and patients at risk within each interval defined by the reported risk time points and numbers at risk. The algorithm was stabilized with refined boundary constraints to avoid invalid negative estimates. Finally, IPD (time-to-event and status) was generated. Accuracy was assessed by root mean square error (RMSE), mean absolute error, maximum absolute error, and Kolmogorov-Smirnov test between reconstructed and original curves.

For this single-arm meta-analysis, continuous variables (e.g., survival time) were summarized as pooled median (IQR) or mean (SD) (no WMD used due to lack of a control group). Dichotomous variables (e.g., objective response rate, adverse events) were analyzed using pooled proportions with 95% CI (RR was not applicable). Medians and IQRs were converted to means and SDs using validated methods when needed. Heterogeneity was assessed via Cochran's Q test and I^2^ statistic; a DerSimonian-Laird random-effects model was used for substantial heterogeneity (*P* < 0.05 or I^2^ > 50%), otherwise a fixed-effects model. For some outcomes, 95% CIs were calculated using the Hartung-Knapp-Sidik-Jonkman method. A two-sided *P* < 0.05 was statistically significant.

## Results

### Search results

**Figure 1:**
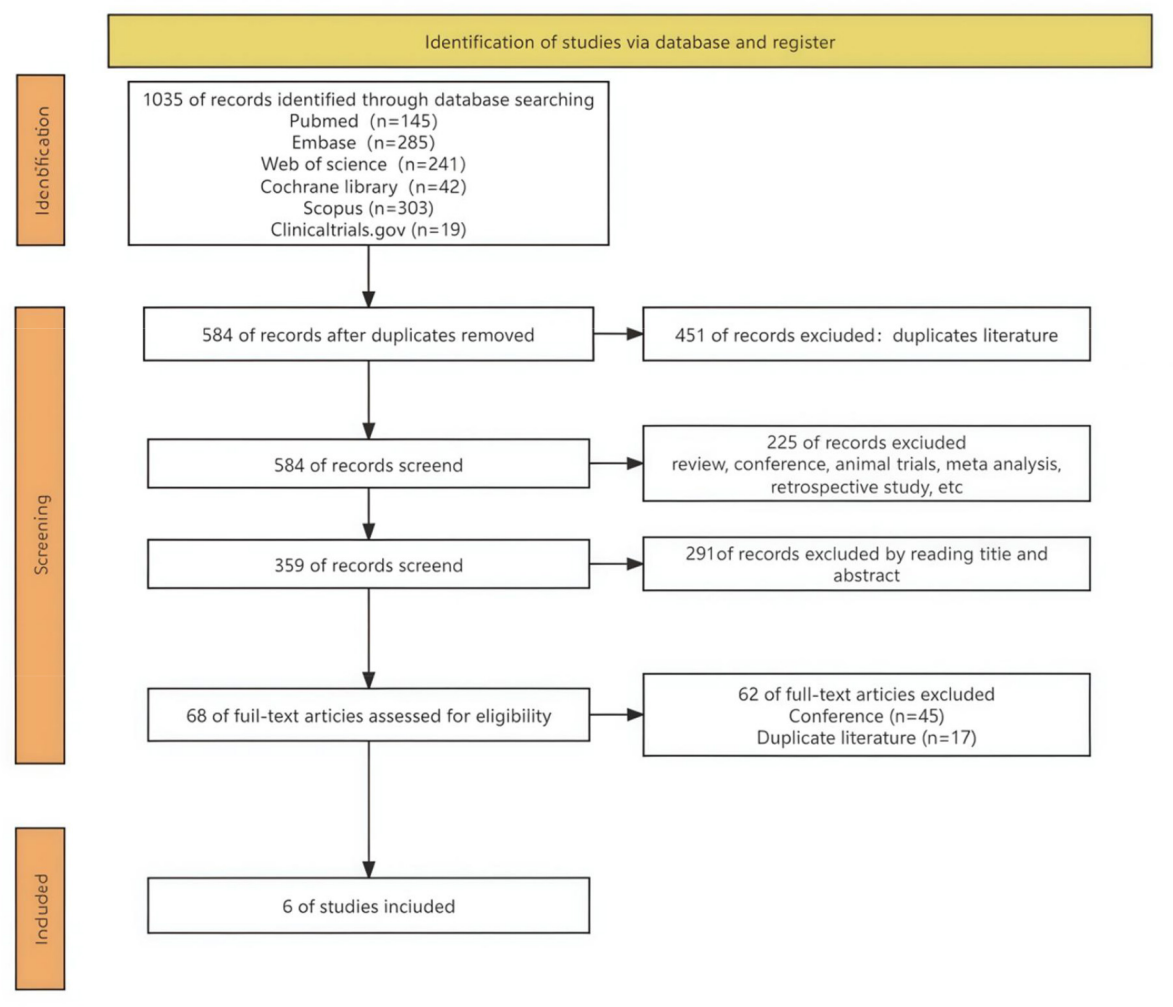
Flow chart of literature search strategies.

Figure 1 illustrates the literature selection process. A total of 1,035 studies were initially identified. After removing 451 duplicate studies primarily via the automatic deduplication function of software, 584 studies remained. Non-eligible study types (e.g., review articles, case reports, animal studies, meta-analyses, and retrospective studies) were excluded, resulting in the elimination of 225 irrelevant publications. Subsequently, 168 articles were further excluded following manual title and abstract screening. Finally, 6 studies were deemed eligible after full-text review, Supplementary material retrieval, and verification of NCT trial registration numbers.

### Patient characteristics and quality assessment

The final analysis included 631 patients who received tebentafusp, derived from a total pool of 850 patients across seven studies (five single-arm trials and one randomized controlled trial). Specifically, data from the tebentafusp cohorts were extracted for this meta-analysis. Study quality was appraised with the modified Jadad scale for RCTs and the MINORS tool for single-arm trials. Comprehensive baseline characteristics and quality assessment results are detailed in Table 1.

**Table 1:** Characteristics of the included studies.

Author, year	Country	Design	NCT	Follow up months	Cases	Total	Age (median, range)	Male	Quality
Middleton (2020)	UK	Single-arm study	NCT01211262	NA	19	84	58.7 (25–78)	54	13
Richard (2022)	USA	Single-arm study	NCT02570308	32.4	42	42	59.0 (39–76)	19	13
Joseph (2024)	UK	Single-arm study	NCT02570308	48.5	146	146	61.0 (25–88)	72	13
Jessica (2023)	UK	RCT	NCT03070392	NA	245	378	65.0 (24–90)	190	4
Ernesto (2025)	Italy	Single-arm study	NA	28	52	52	58.7 (38–87)	27	11
Carvajal (2022)	USA	Single-arm study	NCT02570308	19.5	127	148	61.0 (25–88)	63	13

### Efficacy

**Figure 2:**
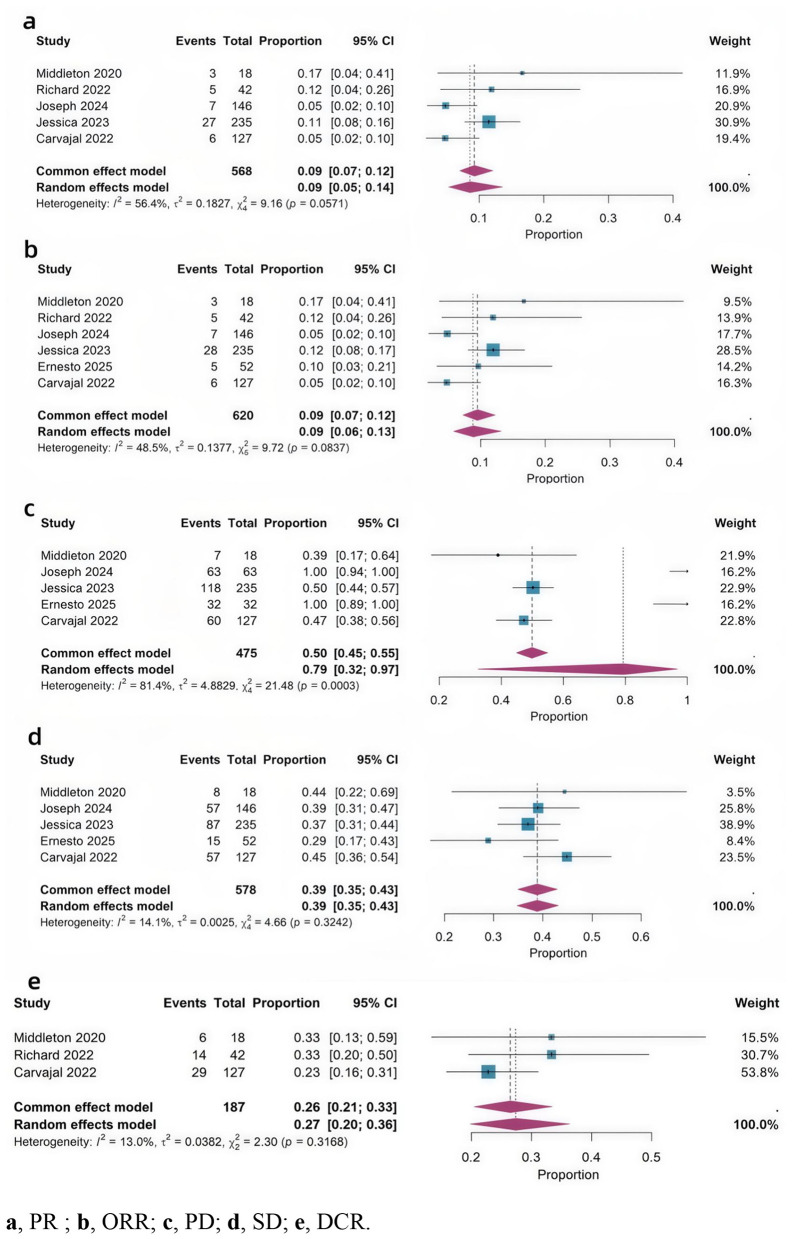
Forest plot of the meta-analysis for PR, ORR, PD, SD, DCR. **(a)**, PR; **(b)**, ORR; **(c)**, PD; **(d)**, SD; **(e)**, DCR.

Table 2 provides a concise summary of efficacy outcomes. The relevant outcomes for metastatic uveal melanoma (mUM) patients treated with tebentafusp are as follows: PR (0.09, 95%CI, 0.05 to 0.14) (Figure 2a), ORR (0.09,95% CI, 0.06 to 0.13) (Figure 2b), PD (0.79, 95% CI, 0.32 to 0.97) (Figure 2c), SD (0.39, 95% CI, 0.35 to 0.43) (Figure 2d), and DCR (0.27, 95% CI, 0.20 to 0.36) (Figure 2e).

**Table 2:** Summary of the forest plot of main results.

Outcomes	No. of study	Patients	Heterogeneity	Overall effect size	95% CI of overall effect
			I^2^(%) *p*-value		
PR	5	568	56.4, 0.0571	0.09	0.05–0.14
ORR	6	620	48.5, 0.0837	0.09	0.06–0.13
SD	5	578	14.1, 0.3242	0.39	0.35–0.43
PD	5	475	81.4, 0.0003	0.79	0.32–0.97
DCR	3	187	13.0, 0.3168	0.27	0.20–0.36

### Safety

The pooled estimate of the patients with any grade Cytokine release syndrome were 0.88 (95% CI, 0.84 to 0.91). And Grade ≥ 3 Cytokine release syndrome was 0.02 (95% CI, 0.01 to 0.06). The Grade ≥ 3 TRAEs rate was found to be 0.41 (95% CI, 0.17 to 0.71) among patients with uveal melanoma who received tebentafusp as treatment. For a any grade TRAEs, the result was 0.98 (95% CI, 0.75 to 1.00). The most prevalent TRAEs were pyrexia 0.83 (95% CI, 0.79 to 0.86), nausea 0.58 (95% CI, 0.42 to 0.73), fatigue 0.54 (95% CI, 0.38 to 0.70), pruritus 0.72 (95% CI, 0.65 to 0.78), chills 0.60 (95% CI, 0.47 to 0.71), dry skin 0.43 (95% CI, 0.25 to 0.63) and hypotension 0.41 (95% CI, 0.36 to 0.45). The detailed results are in Supplementary Figure and Table 3.

**Table 3:** Summary of the forest plot of the other results.

Outcomes	No. of study	Patients	Heterogeneity	Overall effect size	95% CI of overall effect
			I^2^(%) *p*-value		
1-year OS rate	6	763	61.2, 0.0245	0.66	0.60–0.72
Pyrexia	3	414	24.5, 0.2660	0.83	0.79–0.86
Nausea	3	414	86.5, 0.0006	0.58	0.42–0.73
Fatigue	3	414	84.3, 0.0017	0.54	0.38–0.70
Pruritus	3	414	46.9, 0.1518	0.72	0.65–0.78
Chills	3	414	81.3, 0.0047	0.60	0.47–0.71
Dry skin	3	414	89.1, 0.0001	0.43	0.25–0.63
Hypotension	3	518	0.00, 0.3700	0.41	0.36–0.45
A any grade TRAEs	4	466	92.7, < 0.0001	0.98	0.75–1.00
A grade 3 or higher TRAEs	4	466	89.8, < 0.0001	0.41	0.17–0.71
Cytokine release syndrome	3	414	0.00, 0.6457	0.88	0.84–0.91
Grade ≥ 3 Cytokine release syndrome	3	414	44.8, 0.1637	0.02	0.01–0.06

### Overall survival and progression-free-survival

**Figure 3:**
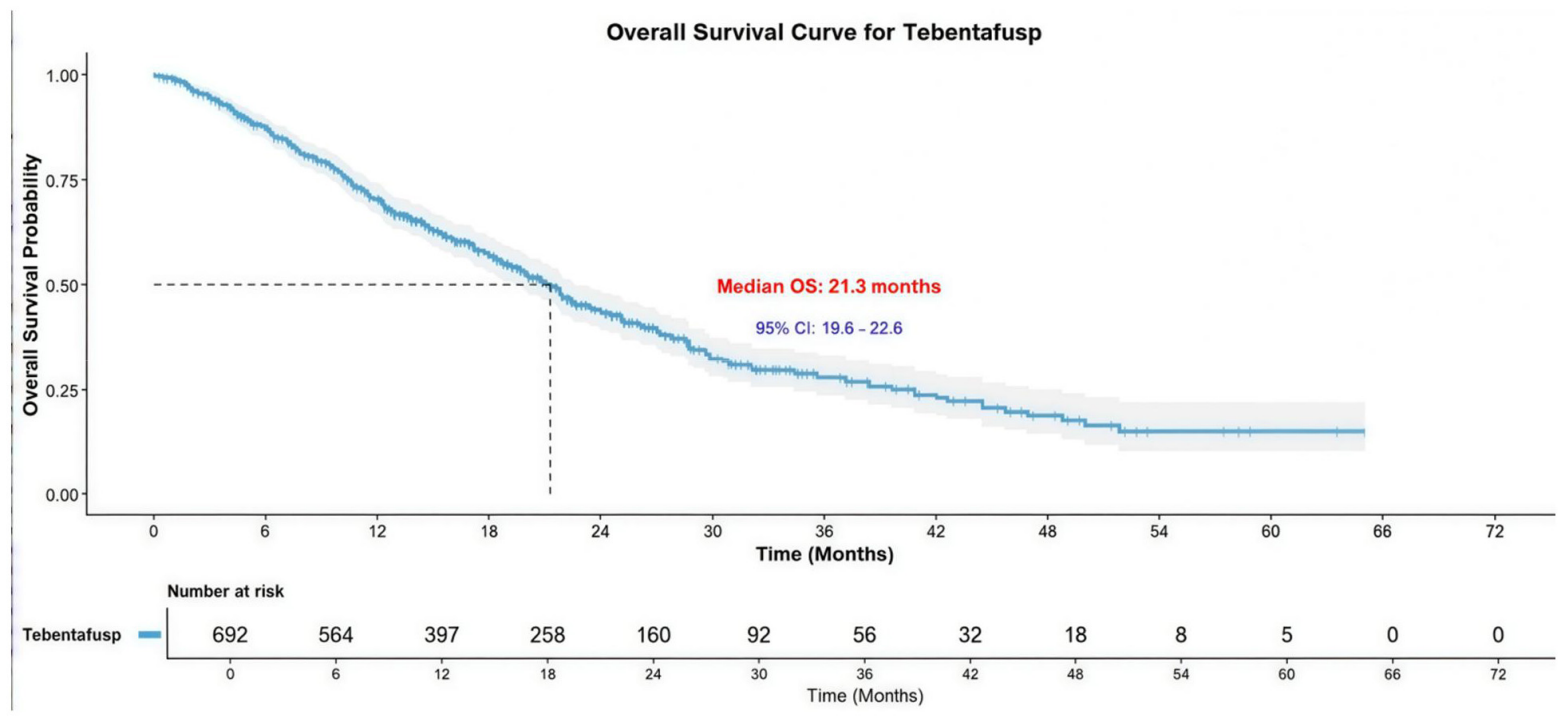
Kaplan-Meier curves for overall survival.

Supplementary Figure 3 and Figure 3 presented the 1-year OS rate and OS results of patients diagnosed with metastatic uveal melanoma, who received tebentafusp as treatment. Following the reconstruction of the cohort, we conducted an additional evaluation of OS using a Kaplan-Meier curve. Significantly, the median OS was 21.3 months. In this meta-analysis, five studies reported survival outcomes one year later, and we analyzed them (0.64, 95% CI, 0.57 to 0.71). As for progression-free survival, five studies have reported the relevant results and median PFS was 3.6 months.

### Sensitivity analyses and publication bias

Sensitivity analyses were performed for the primary outcomes, and carry out the analysis by leave-one-out. For publication bias, we plotted the Doi plot and calculated the LFK index (Luis Furuya-Kanamori index = 0.9). Further details are available in the Supplementary materials.

## Discussion

Our meta-analysis demonstrates that tebentafusp achieves an overall objective response rate (ORR) of 9%, a disease stabilization rate of 39%, and a disease control rate (DCR) of 27%. The 1-year overall survival (OS) rate is 64%, with a median OS (mOS) of 21.3 months and a median progression-free survival (mPFS) of 3.6 months. Regarding safety, the incidence of any-grade adverse events (AEs) is 98%, while that of grade ≥ 3 AEs is 41%. Any-grade cytokine release syndrome (CRS) occurs in 88% of patients, with grade ≥ 3 CRS in 2%. The most common AEs (each >40%) include pyrexia, nausea, fatigue, pruritus, chills, dry skin, and hypotension. Notably, these findings highlight significant AEs and underscore the importance of close monitoring during treatment. Compared with previous meta-analyses (47, 48), the present analysis excludes retrospective studies and case reports, representing the first high-quality meta-analysis to systematically evaluate the safety and efficacy of tebentafusp for metastatic uveal melanoma (mUM). Given its survival benefits alongside a 41% incidence of grade ≥ 3 AEs and only 2% grade ≥ 3 CRS, tebentafusp emerges as a feasible therapeutic option—one that still warrants close attention to safety.

Previous studies have reported that the 1-year OS rate with tebentafusp exceeds 60%, which is broadly consistent with our findings (47, 52, 53). Our mOS results are largely comparable to those from two clinical trials with >2 years of follow-up, both reporting mOS >20 months (52, 54). Notably, most studies have shown that tebentafusp is associated with a relatively high incidence of AEs, including CRS and skin-related adverse reactions (46–48, 52–56). Consistent with these reports, we observed a high AE incidence; however, the incidence of grade ≥3 CRS was relatively low (2%).

Although the pooled incidence of grade ≥3 treatment-related AEs (trAEs) in our meta-analysis is non-negligible (41%), it should be interpreted within the context of tebentafusp's novel mechanism of action and the historically dismal prognosis of mUM. By design, tebentafusp is a T-cell receptor bispecific fusion protein that redirects T cells to gp100-positive cells; the consequent robust immune activation is pharmacologically expected to induce cytokine-mediated and skin-related toxicities rather than off-target, irreversible organ damage (46). Importantly, this safety profile is both predictable and manageable with standardized supportive measures. Consistent with pivotal trial reports, the most frequent trAEs in our analysis were pyrexia (83%), pruritus (72%), chills (60%), nausea (58%), and fatigue (54%)—events that largely align with the “cytokine-mediated (e.g., CRS) and skin-related” toxicity pattern described in tebentafusp AE management guidelines and regulatory labels (46, 57, 58). Although 88% of patients experienced CRS, grade ≥ 3 CRS was uncommon (2% in our pooled estimate), and randomized data indicate that step-up (ramp-up) dosing reduces severe CRS to < 1%, with no grade 4–5 CRS reported (46, 58). These findings underscore that the inflammatory cascade can generally be contained with pre-hydration, close monitoring (especially during the first three infusions), and early intervention (58). Furthermore, expert guidance emphasizes proactive monitoring of liver enzymes and prompt management of transaminase elevations, which are common but typically reversible (58). The predominance of skin-related toxicities (rash, pruritus, dry skin) and laboratory abnormalities reflects mechanism-based targeting of gp100-positive melanocytes rather than irreversible organ toxicity (46). Of note, early rash has been associated with improved outcomes, suggesting that some of these “toxicities” may serve as pharmacodynamic markers of effective immune engagement (46). Against this toxicity profile, the survival benefit conferred by tebentafusp is unprecedented in mUM. Before its approval, real-world series and systematic reviews reported a median OS of approximately 12–13 months, with rapid attrition thereafter (e.g., 52% alive at 1 year and 25% at 2 years) (46); another review noted that OS across conventional modalities commonly ranged from 3 to 12 months (59). By contrast, our meta-analysis demonstrates a median OS of 21.3 months with tebentafusp—a clinically meaningful extension of survival in a disease where effective options have been lacking. Even with a modest ORR (9%), early T-cell activation (manifesting as CRS/skin events) appears to be a mechanistic correlate of long-term benefit rather than merely an adverse effect (46). Taken together, the high rate of grade ≥ 3 trAEs reflects an on-target immune effect that can be mitigated through structured monitoring, step-up dosing, and evidence-based supportive care algorithms (58). Given the historically poor outcomes with prior therapies, the overall risk–benefit balance of tebentafusp remains favorable, supporting its use in eligible patients with careful multidisciplinary management.

### Advantages of this meta-analysis

First, we conducted a comprehensive literature retrieval across multiple authoritative databases, including PubMed, Embase, Web of Science, Scopus, Cochrane Library, and ClinicalTrials.gov, to minimize the risk of missing eligible studies. Additionally, no restrictions on disease types were imposed in the initial search strategy to capture as many tebentafusp-related publications as possible. Following database retrieval, we further adopted a strict multi-round literature screening procedure to identify qualified studies. Such exhaustive database coverage and unrestricted search scope substantially reduced the impact of potential publication bias and ensured the comprehensiveness and representativeness of the included evidence. Second, we excluded low-quality retrospective studies and included only high-quality clinical trials, making this one of the current meta-analyses with high evidence quality. Third, we reconstructed Kaplan-Meier curves for OS using the IPDfromKM software package to present survival outcomes in a clear and interpretable manner. Fourth, we conducted a comprehensive analysis of AEs, including any-grade and grade ≥ 3 CRS as well as any-grade and grade ≥ 3 AEs. Fifth, we performed a preliminary analysis of response duration and PFS.

### Limitations of this meta-analysis

First, most eligible studies were designed as single-arm non-comparative trials, while the only included RCT was restricted to its tebentafusp intervention arm without incorporating a parallel control group. The general absence of synchronized control arms limits direct head-to-head comparison between tebentafusp and standard regimens such as anti-PD-1/CTLA-4 combination therapy. Moreover, cross-study indirect comparisons are hampered by inter-trial heterogeneity in baseline patient profiles, disease burden, prior treatment lines, follow-up duration and inclusion criteria, which may introduce confounding bias, overestimate the genuine therapeutic effect, and fail to reliably clarify the relative merits and demerits of tebentafusp vs. other available treatments. Second, significant heterogeneity may exist across included studies, including differences in baseline patient characteristics (e.g., metastasis burden, prior treatment history, comorbidities), treatment protocols (e.g., dose, duration), and outcome assessment methods (e.g., timing of response evaluation, AE definitions). Although statistical heterogeneity was tested and considered manageable, it may still undermine the robustness of the pooled estimates. Third, this analysis is vulnerable to publication bias. All included studies reported positive efficacy or acceptable safety; no negative or neutral results were included, potentially introducing optimistic bias in efficacy endpoints (e.g., ORR, PFS) and underrepresenting rare or serious AEs. Fourth, limited generalizability is a concern: the included studies may have recruited highly selected populations (e.g., excluding patients with severe organ dysfunction or multiple prior regimens), which may not fully reflect real-world practice, thereby limiting applicability to broader mUM cohorts. Fifth, limitations specific to survival outcome reconstruction from published Kaplan-Meier curves should be noted. Follow-up duration varied substantially across studies (range 19.5–48.5 months), introducing clinical and methodological heterogeneity: shorter follow-up may underestimate long-term events and overestimate early survival, whereas longer follow-up may capture more late events and yield relatively poorer estimates, distorting the pooled survival distribution. Additionally, reconstructing individual patient data from graphical curves inevitably introduces digitization bias, coordinate extraction error, and monotonicity adjustment deviation, even with standardized algorithms—these measurement errors may accumulate and reduce the precision of pooled median survival and survival probability estimates. Inconsistent timing and intensity of follow-up, along with differences in event adjudication and censoring rules across cohorts, may cause informative censoring bias and further compromise comparability of survival outcomes. Moreover, the limited number of studies eligible for IPD reconstruction may amplify small-study bias and selection bias, as only studies with sufficiently clear and complete Kaplan-Meier plots could be included, potentially favoring those with more favorable or comprehensively reported outcomes. Sixth, most included studies did not report follow-up time in sufficient detail, and efficacy/safety data were incomplete, limiting assessment of long-term survival benefits and delayed AEs—critical for guiding clinical decisions in this chronic, life-threatening disease. Seventh, differences in original report quality (e.g., incomplete AE severity records, missing withdrawal reasons, non-standardized efficacy endpoints) may introduce information bias and reduce reliability of aggregated results. Eighth, planned subgroup analyses (e.g., by tebentafusp dosage, patient characteristics, prior treatment) could not be conducted because only six studies were included, yielding insufficient statistical power for robust, representative results. Collectively, these sources of heterogeneity and bias suggest that the pooled survival results should be interpreted with caution, and conclusions should be viewed as hypothesis-generating rather than definitive.

### Impact of high toxicity on real-world practices

The safety profile observed in this meta-analysis underscores a substantial treatment burden: any-grade trAEs occurred in nearly all patients (98%), with grade ≥ 3 events in 41%. The most frequent high-grade events—pyrexia, rash, and hypotension—are hallmarks of tebentafusp's mechanism as an immune-mobilizing bispecific T-cell engager, manifesting largely as cytokine release syndrome. While these events are often described as “manageable” within the protocolized inpatient monitoring and supportive care framework of clinical trials (e.g., intravenous fluids, antipyretics, tocilizumab), their high prevalence fundamentally impacts clinical feasibility and patient selection in real-world practice.

First, the need for intensive monitoring during the initial doses (often requiring hospitalization or prolonged observation) poses a significant logistical and resource challenge, potentially limiting the drug's applicability in healthcare settings without established infrastructure for CRS management. Second, the 41% rate of severe toxicities, particularly hypotension requiring vasopressor support in some cases, demands rigorous patient screening. Clinicians must carefully evaluate candidates for baseline cardiovascular comorbidities, performance status, and frailty, as these factors could predispose patients to poor tolerance and early treatment discontinuation. Consequently, the favorable overall survival benefit attributed to tebentafusp must be weighed against this considerable toxicity burden in a shared decision-making process, especially when palliative alternatives with more established safety profiles exist. Future real-world studies should prospectively evaluate treatment uptake, adherence, and safety outcomes outside the controlled trial environment to better define the patient population deriving net clinical benefit.

## Conclusions

In conclusion, our findings suggest that tebentafusp confers a modest but potentially meaningful survival benefit for metastatic uveal melanoma patients, despite its relatively low objective response rate. Although it is accompanied by a high incidence of adverse events (AEs), these are generally manageable and controllable. Given the limitations of this study, it is critical to conduct additional multicenter randomized controlled trials and expand the sample size to further validate our findings.

## Data Availability

The datasets presented in this study can be found in online repositories. The names of the repository/repositories and accession number(s) can be found in the article/supplementary material.
