# Supplementary material for: Clinical outcomes of tebentafusp in metastatic uveal melanoma: a systematic review and single-arm meta-analysis
Source: Front Med (Lausanne). 2026 Jun 17;13:1826626. doi: 10.3389/fmed.2026.1826626 (PMC13318776; doi:10.3389/fmed.2026.1826626)
Supplement: Supplementary file 1 [file Table_1.docx]

**Supplement material eFigure 1.** Forest plot of the meta-analysis for Cytokine release syndromeand Treatment-Related Adverse Events.

**Supplement material eFigure 2.** Forest plot of the meta-analysis for common Treatment-Related Adverse Events.

**Supplement material eFigure 3.** Forest plot of the meta-analysis for 1-year OS rate.

**Supplementary material eTable1.**Literature search.

**Corresponding fit statistics** (RMSE, MAE, MaxAE, and KS pvalue). Each reconstructed survival curve.

**Quality:**Detailed reports

**Sensitivity analysis:**The primary outcomes

**Publication bias**

**Patient flow diagram**

**Supplement material eFigure 1.** Forest plot of the meta-analysis for Cytokine release syndromeand Treatment-Related Adverse Events.


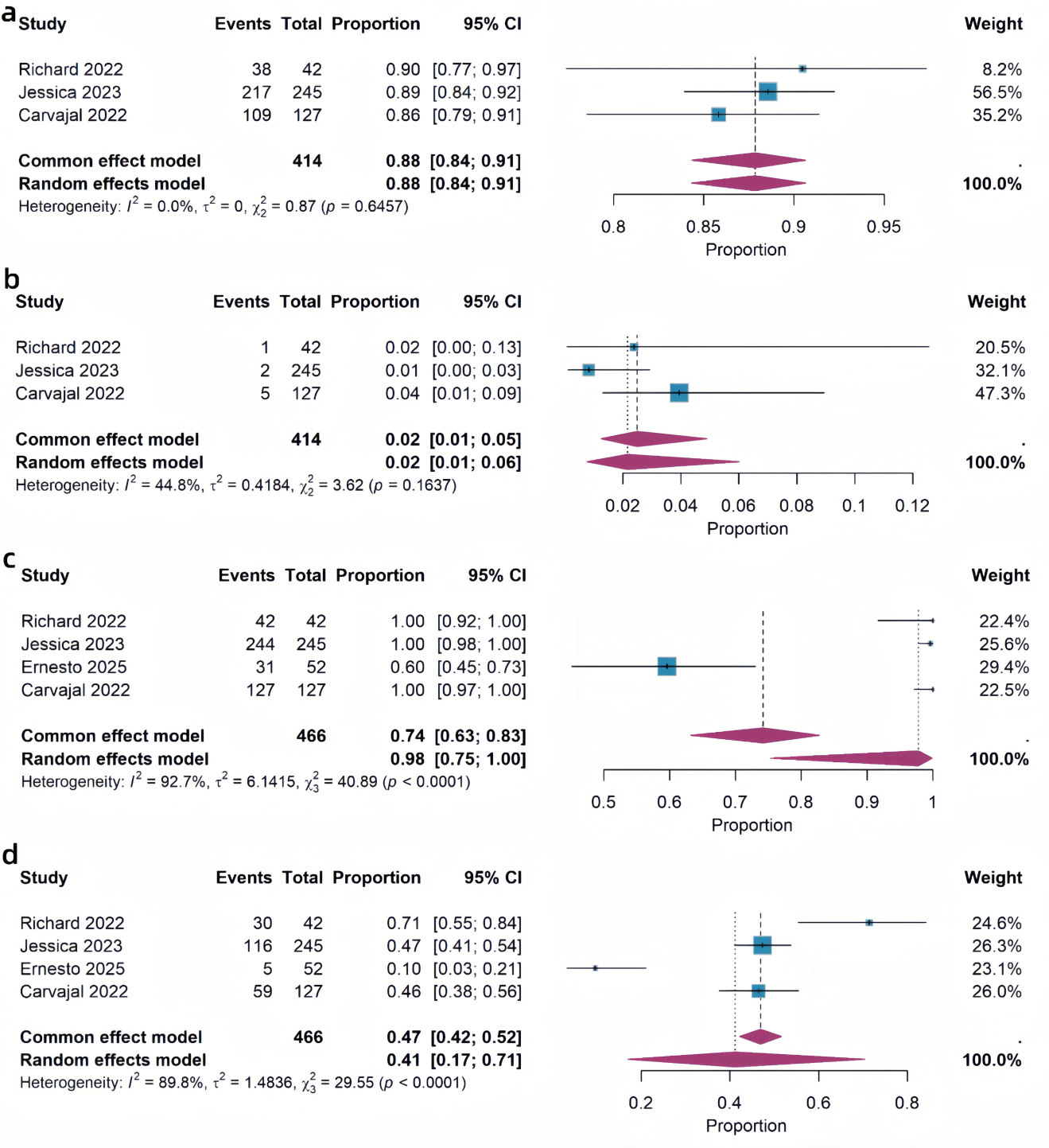


Supplement material eFigure 1a: any grade Cytokine release syndrome; Supplement material eFigure 1b: Grade≥3 Cytokine release syndrome; Supplement material eFigure 1c: any grade TRAEs;Supplement material eFigure 1d: Grade≥ 3 TRAEs rate

**Supplement material eFigure 2.** Forest plot of the meta-analysis for common Treatment-Related Adverse Events.


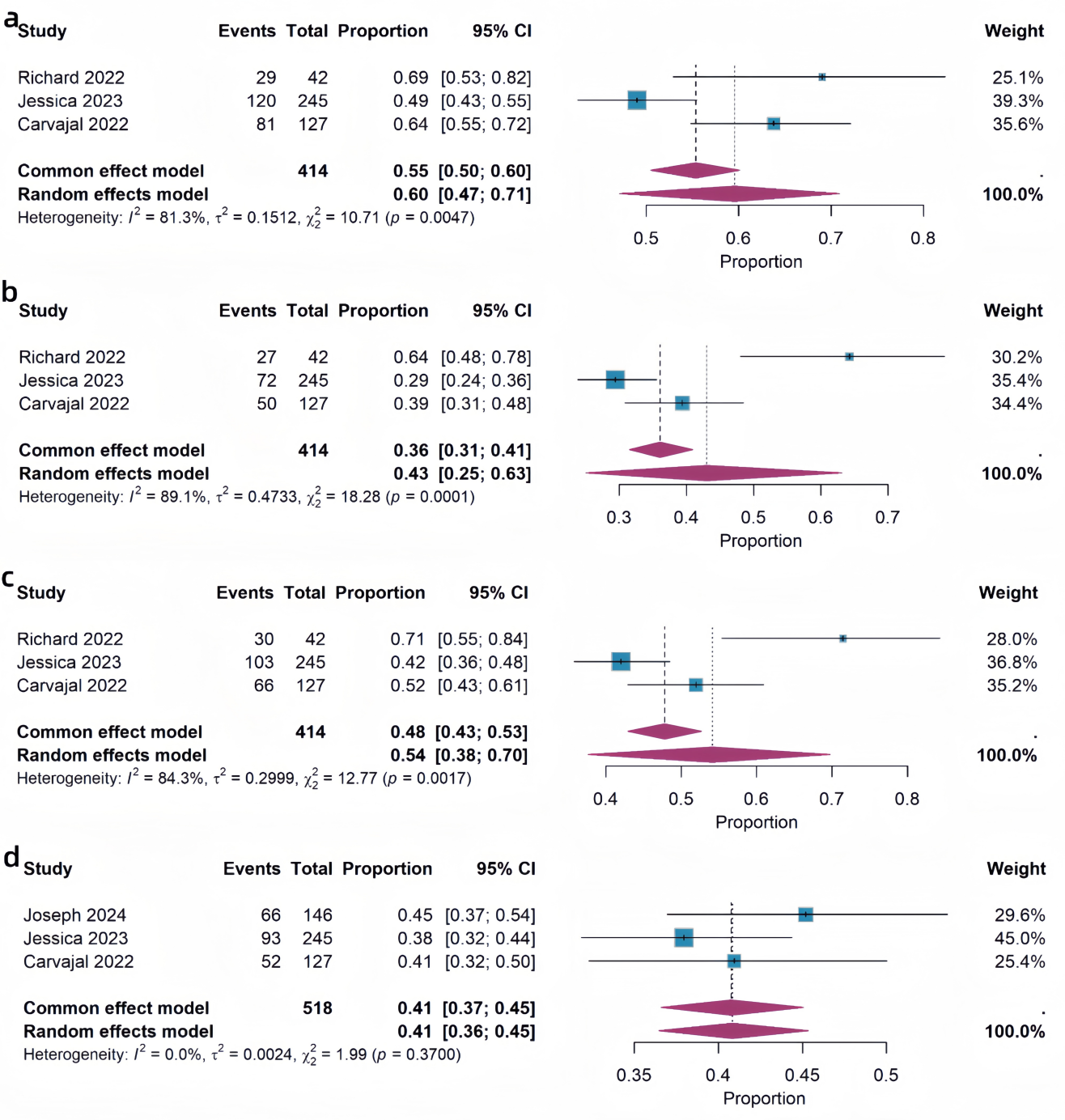


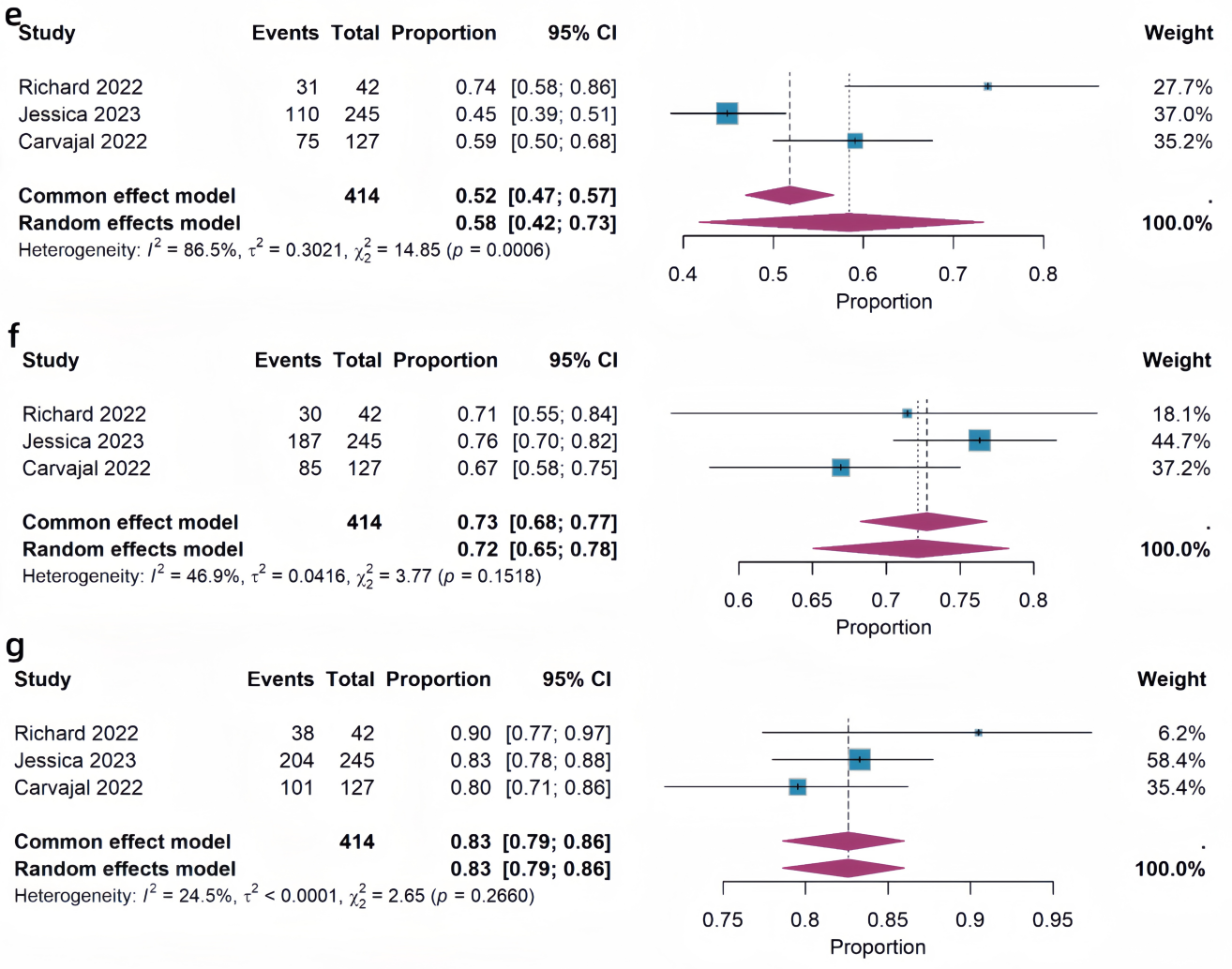


Supplement material eFigure 2a: chills; Supplement material eFigure 2b: dry skin; Supplement material eFigure 2c: fatigue; Supplement material eFigure 2d: hypotension; Supplement material eFigure 2e: nausea; Supplement material eFigure 2f: pruritus; Supplement material eFigure 2g: pyrexia

**Supplement material eFigure 3.** Forest plot of the meta-analysis for 1-year OS rate.


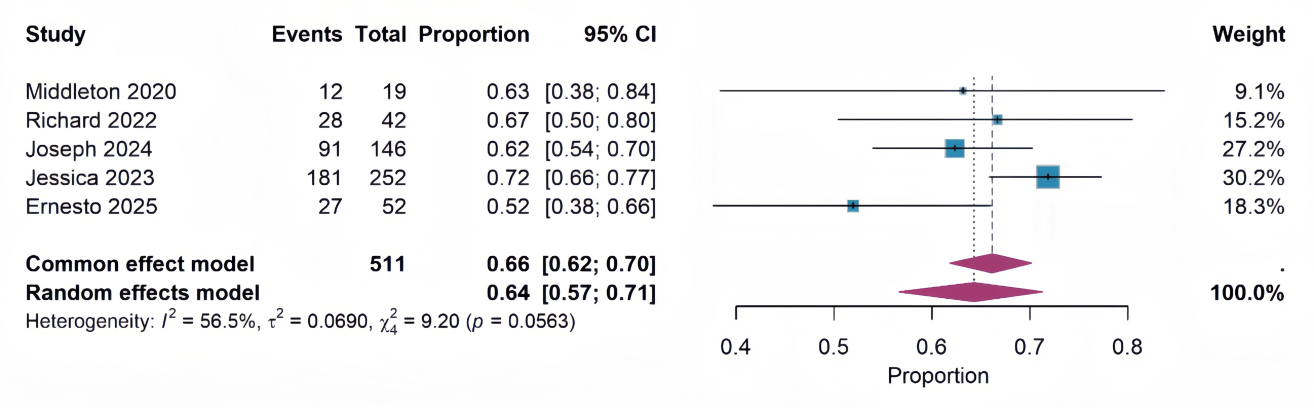


**Supplementary material eTable1.**Literature search

clinicaltrials.gov

Intervention：tebentafusp 19

| PubMed | | |
| --- | --- | --- |
| No. | Query | Results |
| #1 | tebentafusp[Title/Abstract] | 145 |
|  |  |  |

| cochrane library | | |
| --- | --- | --- |
| No. | Query | Results |
| #1 | tebentafusp:ab,ti,kw | 42 |
|  |  |  |

| Embase | | |
| --- | --- | --- |
| No. | Query | Results |
| #1 | tebentafusp:ti,ab,kw | 285 |
|  |  |  |

| Web of science | | |
| --- | --- | --- |
| No. | Query | Results |
| #1 | TS=tebentafusp | 241 |
|  |  |  |

| Scopus | | |
| --- | --- | --- |
| No. | Query | Results |
| #1 | tebentafusp:ti,ab,kw | 303 |
|  |  |  |

**Corresponding fit statistics** (RMSE, MAE, MaxAE, and KS pvalue). Each reconstructed survival curve.

**A** Clinical and molecular response to tebentafusp in previously treated patients with metastatic uveal melanoma: a phase 2 trial


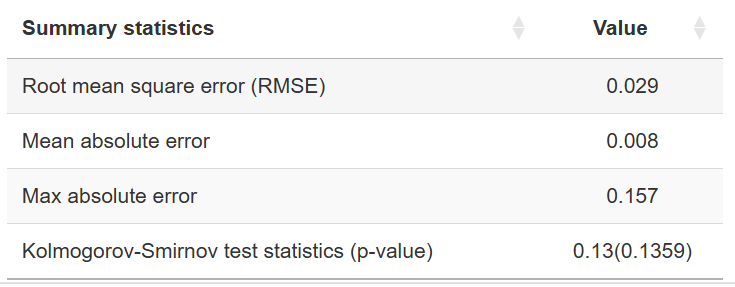


**B** Long-term survival follow-up for tebentafusp in previously treated metastatic uveal melanoma


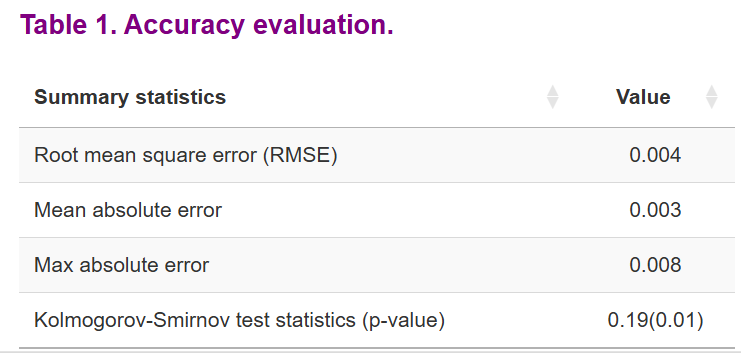


**C** Phase I Study of Safety, Tolerability, and Efficacy of Tebentafusp Using a Step-Up Dosing Regimen and Expansion in Patients With Metastatic Uveal Melanoma


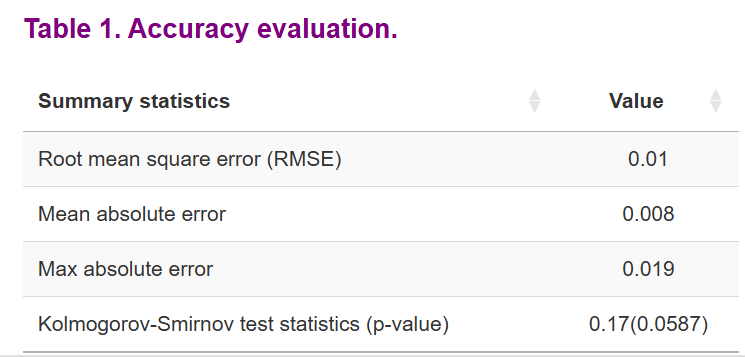


**D** Three-Year Overall Survival with Tebentafusp in Metastatic Uveal Melanoma


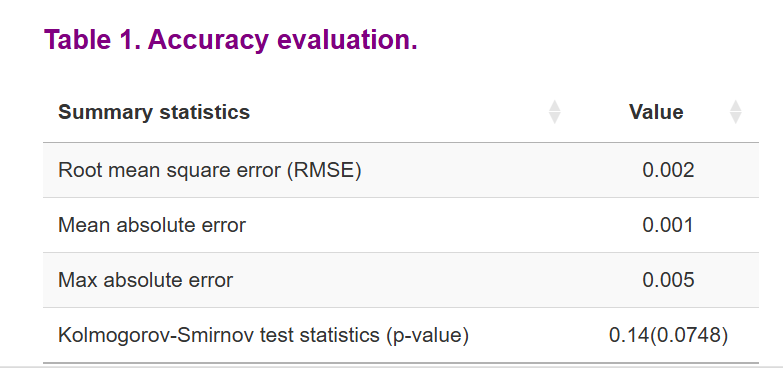


**E** Tebentafusp, A TCR/Anti-CD3 Bispecific Fusion Protein Targeting gp100, Potently Activated Antitumor Immune Responses in Patients with Metastatic Melanoma


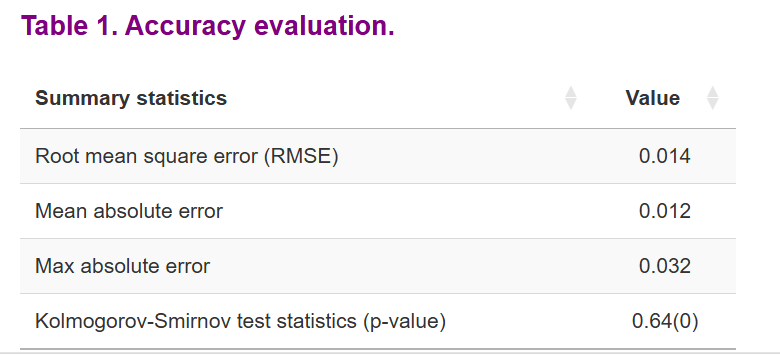


**Quality:**Detailed reports

**Modified Jadad Scale**

**1.Random sequence generation**

Random allocation was clearly used; this was an international multicenter phase III RCT. The randomization method was appropriate.

**2.Allocation concealment**

Random allocation was mentioned, but the concealment method was not specifically described; judged as unclear.

**3.Blinding**

The trial had an open‑label design; no blinding was implemented. Blinding was inappropriate.

**4.Attrition (withdrawals and dropouts)**

The numbers and reasons for withdrawals/dropouts were clearly reported for both groups.

| **Author,year** | **Research type** | **NCT** | **1** | **2** | **3** | **4** | **score** |
| --- | --- | --- | --- | --- | --- | --- | --- |
| Jessica C. Hassel 2023 | RCT | NCT03070392 | 2 | 1 | 0 | 1 | 4 |

**Scale: MINORS (Methodological Index for Non‑Randomized Studies)**

**Scoring rules:**

0 = not reported; 1 = reported but insufficient information; 2 = reported and sufficient information.

Single‑arm studies: only items 1–8 are evaluated; maximum total score = 16.

**Quality classification:**

≥12 points = high quality

8–11 points = moderate quality

<8 points = low quality

| **Author,year** | **Research type** | **NCT** | **1** | **2** | **3** | **4** | **5** | **6** | **7** | **8** | **score** |
| --- | --- | --- | --- | --- | --- | --- | --- | --- | --- | --- | --- |
| Middleton 2020 | single arm | NCT01211262 | 2 | 2 | 2 | 2 | 2 | 2 | 1 | 0 | **13** |
| Carvajal 2022 | single arm、  I phase | NCT02570308 | 2 | 2 | 2 | 2 | 2 | 2 | 1 | 0 | **13** |
| Joseph J Sacco 2024 | single arm | NCT02570308 | 2 | 2 | 2 | 2 | 2 | 2 | 1 | 0 | **13** |
| Rossi 2025 | single arm | NA | 2 | 2 | 1 | 2 | 2 | 2 | 0 | 0 | **11** |
| Carvajal 2022 | single arm、  II phase | NCT02570308 | 2 | 2 | 2 | 2 | 2 | 2 | 1 | 0 | **13** |

**Middleton 2020 NCT01211262**

1. A clearly stated aim

Score: 2

Justification: The aim is clearly described as evaluating the safety, tolerability, efficacy, and mechanism of action of tebentafusp in advanced melanoma. The target population, intervention, and outcomes are well defined.

2. Inclusion of consecutive patients

Score: 2

Justification: Inclusion criteria (HLA‑A2+, advanced melanoma, ECOG ≤1, etc.) are clearly reported. Eligible patients were enrolled consecutively during the study period, with no evidence of selection bias.

3. Prospective collection of data

Score: 2

Justification: Data collection followed a pre‑specified clinical study protocol. Efficacy, safety, and biomarker data were collected as planned, with no post‑hoc modifications.

4. Endpoints appropriate to the aim of the study

Score: 2

Justification: The primary endpoint was safety/MTD; secondary endpoints included ORR, OS, and biomarker changes. These are highly consistent with the study aim, and the intention‑to‑treat (ITT) principle was applied.

5. Unbiased assessment of the study endpoint

Score: 2

Justification: Efficacy was objectively assessed using RECIST 1.1; AEs were standardized with CTCAE 4.0. Biomarkers were evaluated using objective methods such as flow cytometry, Luminex, and IHC.

6. Follow‑up period appropriate to the aim

Score: 2

Justification: Long‑term OS (maximum follow‑up >4 years) was reported, with 1‑year OS as a key observation. The follow‑up duration was sufficient to evaluate survival and duration of response.

7. Loss to follow‑up less than 5%

Score: 1

Justification: Follow‑up and survival data were clearly reported, but the overall loss‑to‑follow‑up rate was not precisely provided. Although no major bias from attrition is suspected, the information is insufficient.

8. Prospective calculation of the study size

Score: 0

Justification: This was a phase I/II dose‑finding study; no sample size calculation was reported. Parameters such as α, β, and effect size were not provided.

**Carvajal 2022 NCT02570308**

1. A clearly stated aim

Score: 2

Justification: The aim is clearly described as determining the RP2D of tebentafusp with step‑dosing, and evaluating safety, pharmacokinetics/pharmacodynamics, and preliminary efficacy. The target population, intervention, and endpoints are fully and clearly defined.

2. Inclusion of consecutive patients

Score: 2

Justification: Inclusion/exclusion criteria are clearly listed. Eligible patients were enrolled consecutively during the study period, with no evidence of selective enrollment bias.

3. Prospective collection of data

Score: 2

Justification: Data collection followed a pre‑specified clinical trial protocol. Safety, efficacy, and biomarker data were collected as planned, with no post‑hoc modifications.

4. Endpoints appropriate to the aim of the study

Score: 2

Justification: The primary endpoints were safety, DLT, and RP2D; secondary endpoints included ORR, OS, PFS, DOR, and biomarkers. These are highly consistent with the objectives of a phase I study.

5. Unbiased assessment of the study endpoint

Score: 2

Justification: Efficacy was assessed using RECIST 1.1, and adverse events using CTCAE 4.03 – both objective criteria. Biomarkers were evaluated using objective methods such as flow cytometry, IHC, and Luminex.

6. Follow‑up period appropriate to the aim

Score: 2

Justification: Median follow‑up was 32.4 months. Long‑term OS, 1‑year OS, and duration of response were reported. The follow‑up duration was sufficient to assess survival endpoints in advanced cancer.

7. Loss to follow‑up less than 5%

Score: 1

Justification: Survival data and censoring information were reported, but the overall loss‑to‑follow‑up / withdrawal rate was not explicitly provided. Information is insufficient to confirm whether it was <5%.

8. Prospective calculation of the study size

Score: 0

Justification: This was a phase I dose‑finding study; no sample size calculation was reported. Parameters such as α, β, effect size, and power were not provided.

**Joseph J Sacco 2024 NCT02570308**

1. A clearly stated aim

Score: 2

Justification: The aim is clearly described as reporting the long‑term survival outcomes of tebentafusp in previously treated metastatic uveal melanoma. The target population, intervention, and observed endpoints are complete and specific.

2. Inclusion of consecutive patients

Score: 2

Justification: Inclusion/exclusion criteria are clearly listed. Eligible patients were enrolled consecutively, with no evidence of selective enrollment bias. Baseline characteristics are clearly presented.

3. Prospective collection of data

Score: 2

Justification: Data collection followed a pre‑specified clinical trial protocol. Efficacy, safety, and biomarker (ctDNA, gp100) data were collected as planned, with no post‑hoc modifications.

4. Endpoints appropriate to the aim of the study

Score: 2

Justification: The primary endpoint was long‑term OS; secondary endpoints included ORR, duration of response, safety, and biomarker associations. These are highly consistent with the study aim.

5. Unbiased assessment of the study endpoint

Score: 2

Justification: Efficacy was assessed using RECIST 1.1, adverse events using CTCAE 4.03, and survival analysis using Kaplan–Meier. ctDNA and IHC are both objective detection methods.

6. Follow‑up period appropriate to the aim

Score: 2

Justification: Median follow‑up was as long as 48.5 months. 1‑, 2‑, 3‑, and 4‑year OS rates were reported. The follow‑up duration fully meets the requirements for long‑term survival evaluation in advanced cancer.

7. Loss to follow‑up less than 5%

Score: 1

Justification: Long‑term survival and censoring information were reported, but the overall loss‑to‑follow‑up / withdrawal rate was not explicitly provided. Information is insufficient to confirm whether it was <5%.

8. Prospective calculation of the study size

Score: 0

Justification: This is a long‑term follow‑up analysis of a phase I/II study; no sample size calculation was reported. Parameters such as α, β, and effect size were not provided.

**Rossi 2025**

1. A clearly stated aim

Score: 2

Justification: The aim is clearly described as evaluating the efficacy and safety of tebentafusp in patients with metastatic uveal melanoma in a real‑world EAP population. The target population, intervention, and outcomes are well defined.

2. Inclusion of consecutive patients

Score: 2

Justification: All patients in the Italian EAP who received tebentafusp were included. There was no selective enrollment, and consecutiveness was good.

3. Prospective collection of data

Score: 1

Justification: No pre‑specified study protocol was mentioned. Information is insufficient.

4. Endpoints appropriate to the aim of the study

Score: 2

Justification: The primary endpoints were OS and PFS; secondary endpoints included ORR, clinical benefit rate, and safety. These are consistent with the objective of a real‑world efficacy evaluation.

5. Unbiased assessment of the study endpoint

Score: 2

Justification: Efficacy was assessed using RECIST criteria, and adverse events were graded according to standard CTCAE. The endpoint assessment is objective.

6. Follow‑up period appropriate to the aim

Score: 2

Justification: Median follow‑up was 28 months, which is sufficient to evaluate long‑term outcomes such as OS and PFS in advanced melanoma.

7. Loss to follow‑up less than 5%

Score: 0

Justification: Loss to follow‑up, censoring, and withdrawal were not mentioned at all. Information is missing, making judgment impossible.

8. Prospective calculation of the study size

Score: 0

Justification: No sample size calculation was performed.

**Carvajal 2022 NCT02570308**

1. A clearly stated aim

Score: 2

Justification: The aim is clearly described as evaluating the objective response rate, safety, OS, PFS, and biomarker associations of tebentafusp in previously treated metastatic uveal melanoma. The target population, intervention, and endpoints are complete and明確.

2. Inclusion of consecutive patients

Score: 2

Justification: Inclusion/exclusion criteria are clearly defined. Eligible patients were enrolled consecutively, with no selective enrollment bias. This was a multicenter international cohort.

3. Prospective collection of data

Score: 2

Justification: Data collection followed a pre‑specified clinical trial protocol. Efficacy, safety, and ctDNA data were collected as planned, with no post‑hoc modifications.

4. Endpoints appropriate to the aim of the study

Score: 2

Justification: The primary endpoint was ORR; secondary endpoints included OS, PFS, DCR, safety, and ctDNA associations. These are highly consistent with the objectives of a phase II study.

5. Unbiased assessment of the study endpoint

Score: 2

Justification: Efficacy was assessed using RECIST 1.1 (independent blinded central review), AEs using CTCAE 4.03, and survival using Kaplan–Meier. The endpoint assessment is objective and standardized.

6. Follow‑up period appropriate to the aim

Score: 2

Justification: Median follow‑up was 19.5 months, and 1‑ and 2‑year OS rates were reported. The follow‑up duration was sufficient to evaluate survival outcomes in advanced cancer.

7. Loss to follow‑up less than 5%

Score: 1

Justification: Survival censoring information was reported, but the overall loss‑to‑follow‑up / withdrawal rate was not explicitly provided. Information is insufficient to confirm whether it was <5%.

8. Prospective calculation of the study size

Score: 0

Justification: Only a planned enrollment target was described; no formal sample size calculation, α, β, or effect size was reported.

**Sensitivity analysis:**The primary outcomes


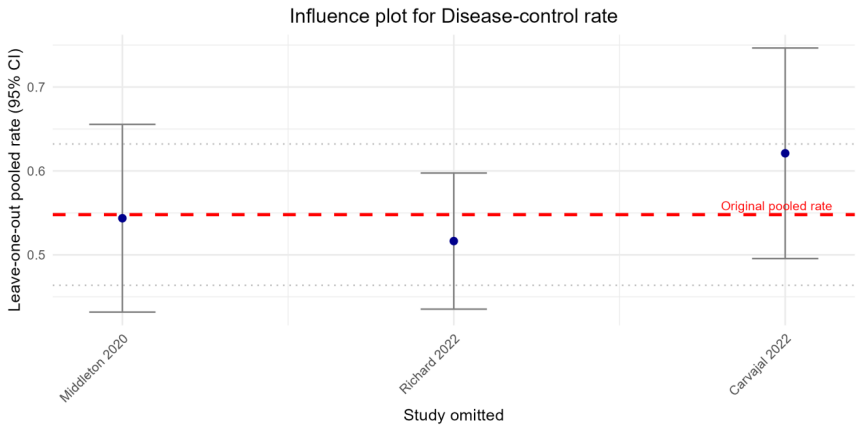


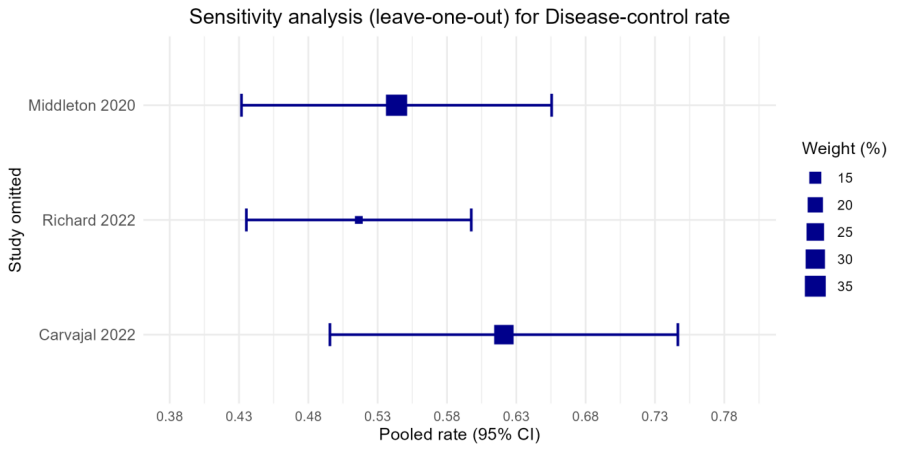


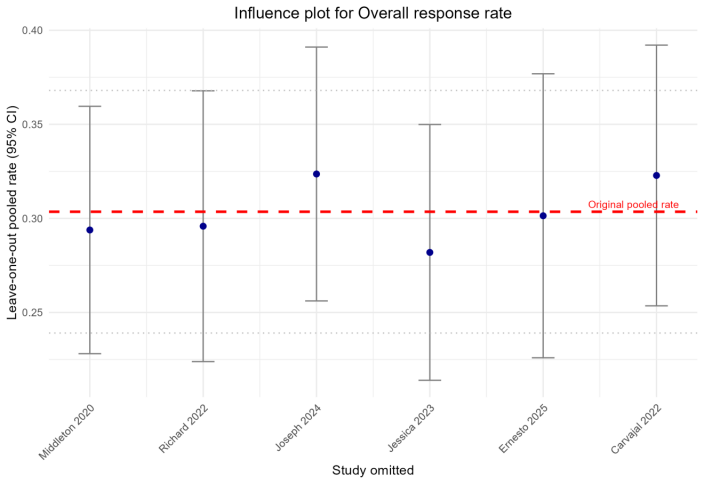


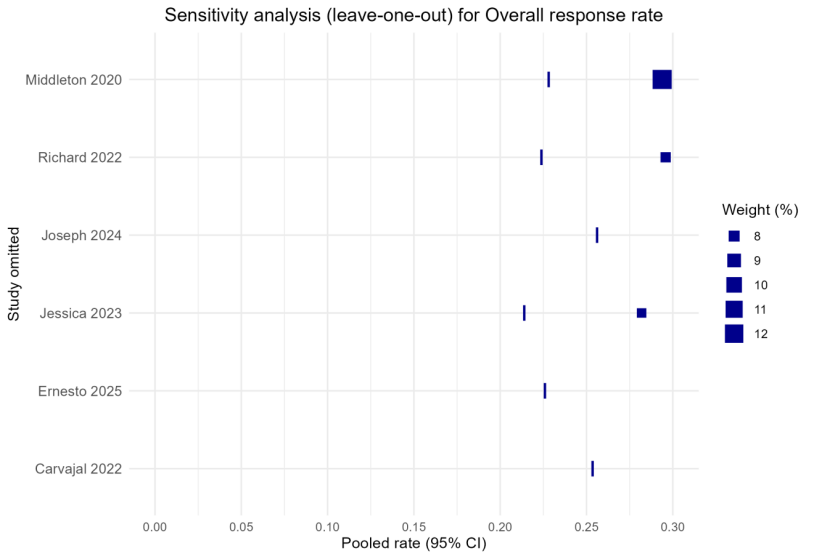


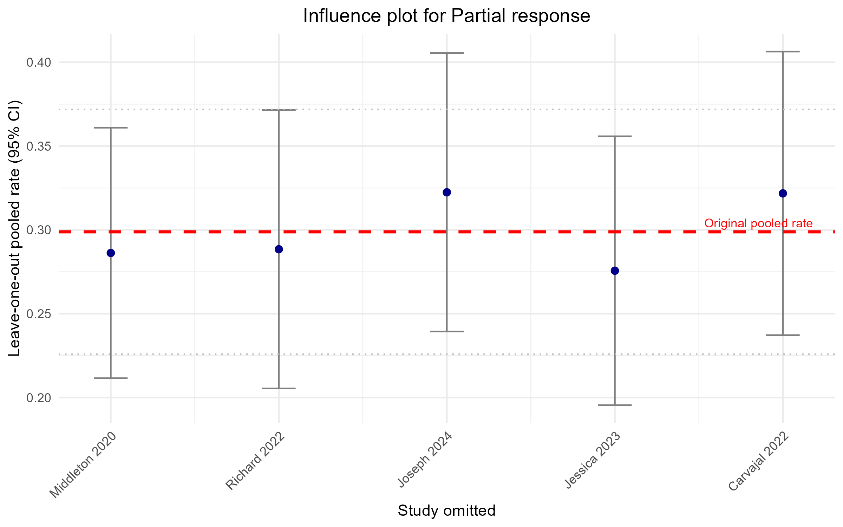


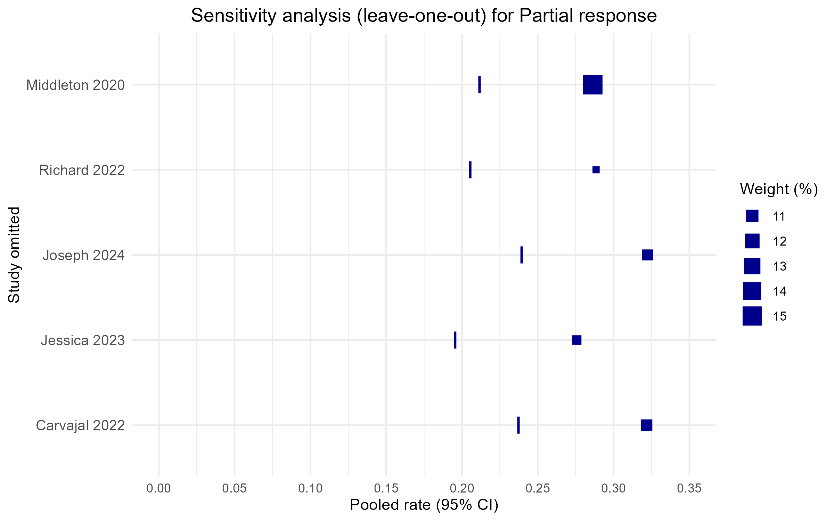


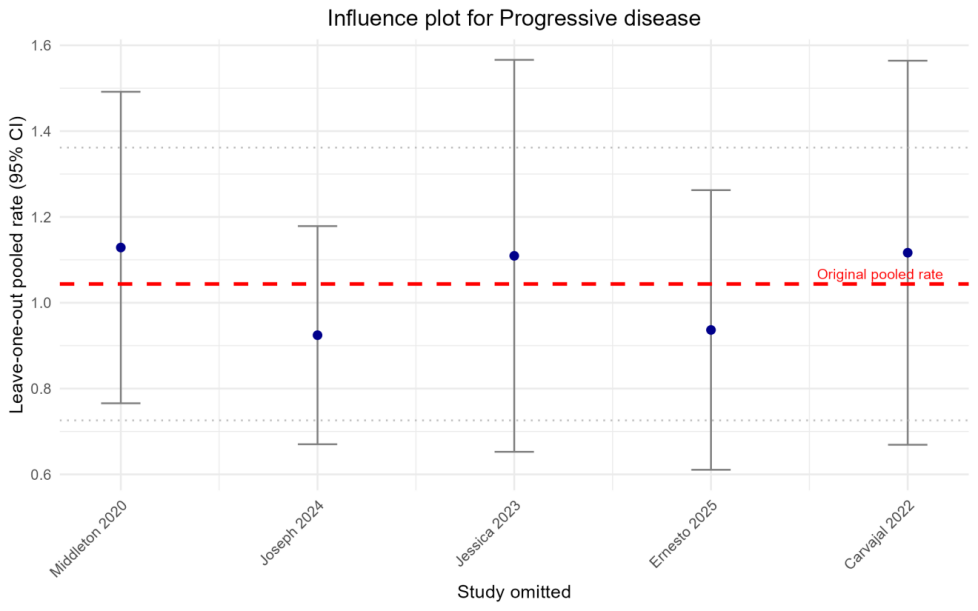


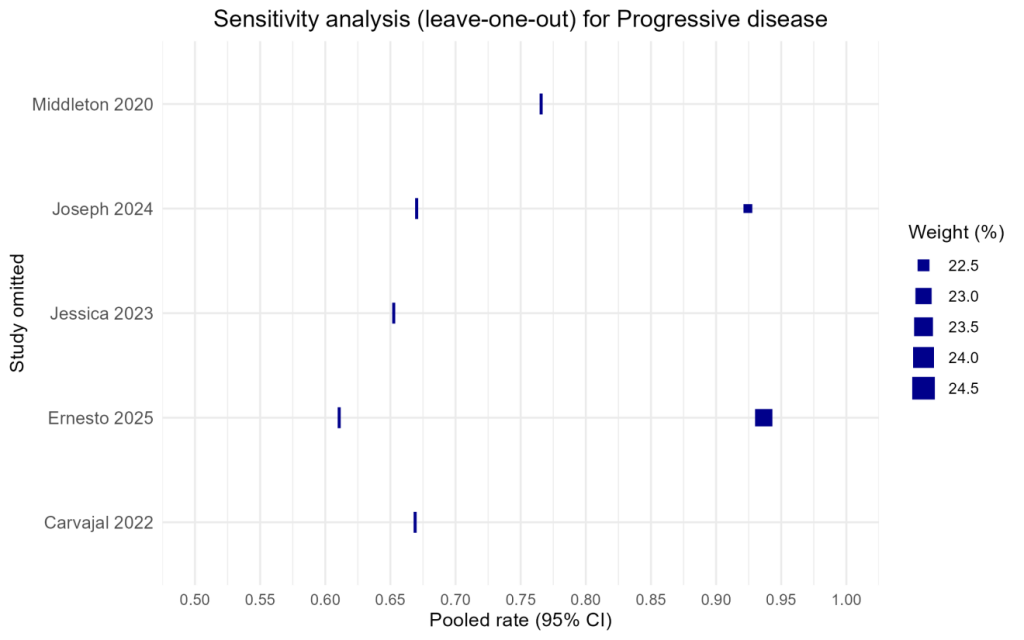


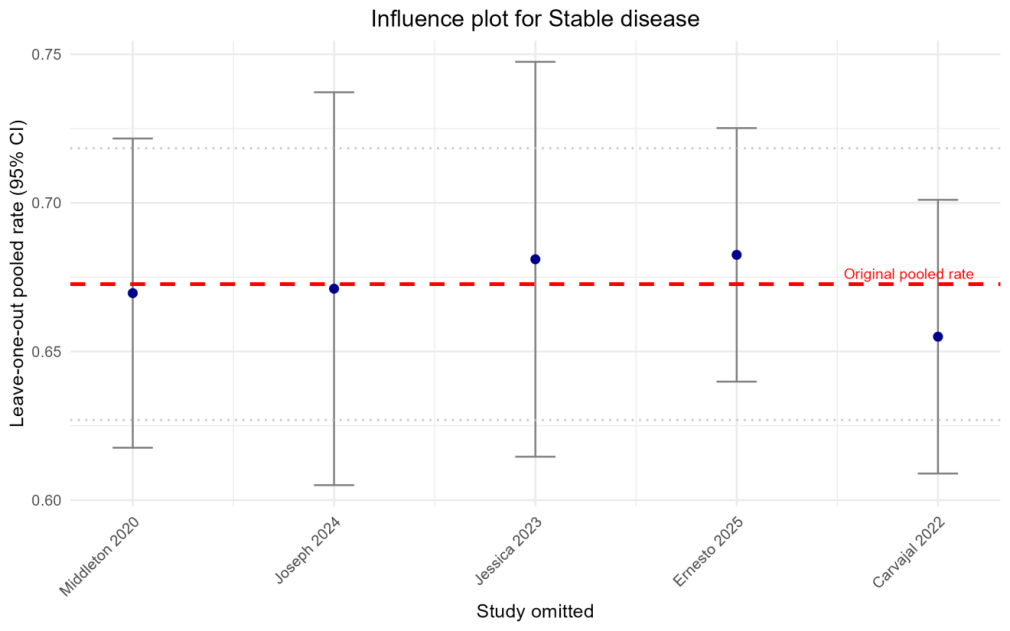


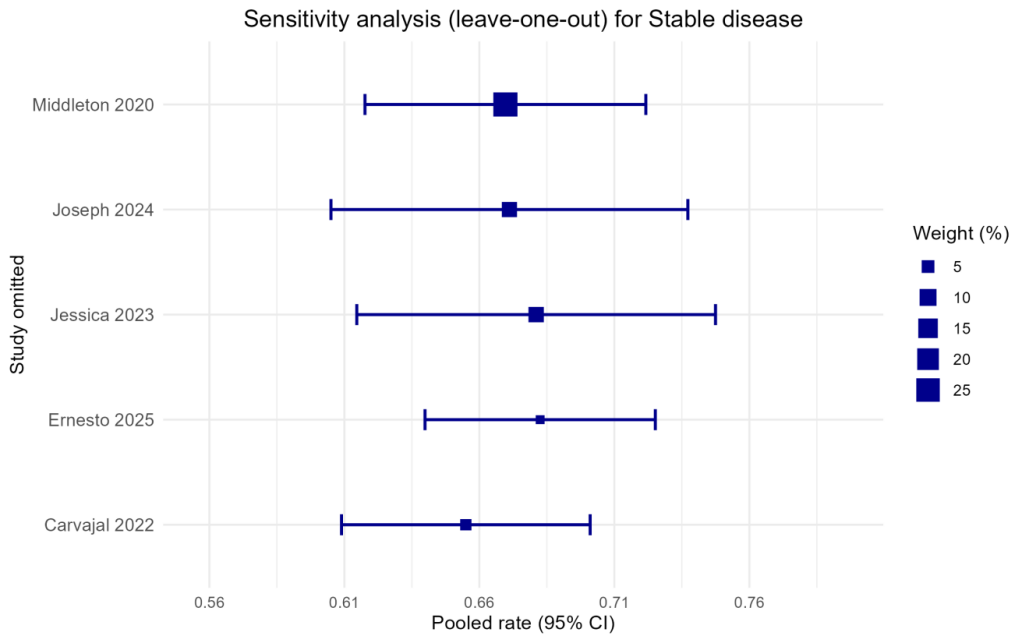


**Publication bias**


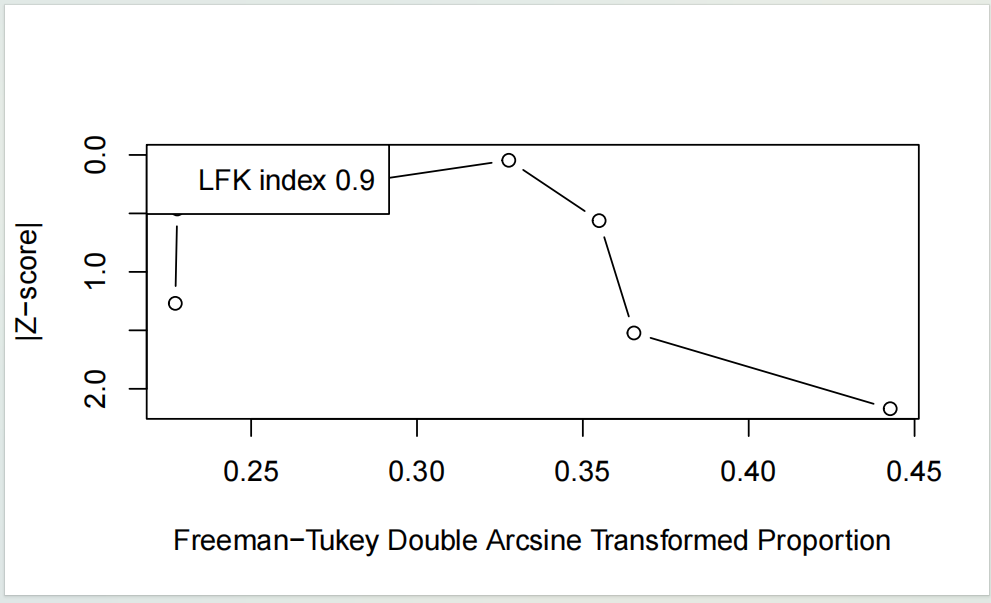


**Patient flow diagram**

Total screened across 6 eligible studies

↓

1. Middleton 2020

Initial screening: 84 patients (HLA-A2+ advanced melanoma)

↓

Exclusions: No documented exclusion

↓

Final analysed population: 84 patients

(cutaneous melanoma=61; **uveal melanoma=19**; other subtypes=4)

2. Richard 2022

Initial screening: 42 patients (HLA-A*02:01+ metastatic uveal melanoma)

↓

Exclusions: 0 patients

↓

Final analysed population: 42 patients

3. Joseph 2024

Initial screening: 146 patients

↓

Exclusions: 0 patients (all received ≥1 dose of study drug)

↓

Final analysed population: 146 patients

4. Jessica 2023

Initial randomisation: 378 patients

↓

Untreated exclusions:

Tebentafusp group: 7 untreated

Control group: 15 untreated

↓

ITT analysis population: 378 patients

↓

Safety analysis population: 356 patients (245 + 111)

5. Ernesto 2025

Initial enrollment: 52 patients

↓

Exclusions: 0 patients

↓

Final analysed population: 52 patients

6. Carvajal 2022

Initial screening: 148 patients (HLA-A*02:01+)

↓

Exclusions: 21 patients (failed inclusion/exclusion criteria)

↓

Final analysed population: 127 patients
